# Distance and force visualisations for improved simulation of intracranial aneurysm clipping

**DOI:** 10.1007/s11548-021-02413-1

**Published:** 2021-05-29

**Authors:** Mareen Allgaier, Belal Neyazi, Bernhard Preim, Sylvia Saalfeld

**Affiliations:** 1 Faculty of Computer Science, Otto-von-Guericke University Magdeburg, Universitätsplatz 2, 39106 Magdeburg, Germany; 2 University Hospital Magdeburg, Leipziger Str. 44, 39120 Magdeburg, Germany; 3 Forschungscampus STIMULATE, Magdeburg, Germany

**Keywords:** Aneurysm clipping simulation, Surgical training, Distance visualisation, Force visualisation

## Abstract

**Purpose:**

The treatment of cerebral aneurysms shifted from microsurgical to endovascular therapy. But for some difficult aneurysm configurations, e.g. wide neck aneurysms, microsurgical clipping is better suited. From this combination of limited interventions and the complexity of these cases, the need for improved training possibilities for young neurosurgeons arises.

**Method:**

We designed and implemented a clipping simulation that requires only a monoscopic display, mouse and keyboard. After a virtual craniotomy, the user can apply a clip at the aneurysm which is deformed based on a mass–spring model. Additionally, concepts for visualising distances as well as force were implemented. The distance visualisations aim to enhance spatial relations, improving the navigation of the clip. The force visualisations display the force acting on the vessel surface by the applied clip. The developed concepts include colour maps and visualisations based on rays, single objects and glyphs.

**Results:**

The concepts were quantitatively evaluated via an online survey and qualitatively evaluated by a neurosurgeon. Regarding force visualisations, a colour map is the most appropriate concept. The necessity of distance visualisations became apparent, as the expert was unable to estimate distances and to properly navigate the clip. The distance rays were the only concept supporting the navigation appropriately.

**Conclusion:**

The easily accessible surgical training simulation for aneurysm clipping benefits from a visualisation of distances and simulated forces.

## Introduction

*Unruptured cerebral aneurysms* are pathologic dilatations of blood vessels in the brain. The main danger of cerebral aneurysms is rupture, resulting in a critical bleeding [2]. Because of the severe consequences of a rupture, the risks of not treating the aneurysms have to be weighted against the risks of treatment [4]. Aneurysms can either be treated minimally invasively or via surgical clipping [43]. During the clipping procedure, first a *craniotomy* is performed [17]. Afterwards, the aneurysm is dissected and exposed. Then, clips can be placed carefully at the aneurysm neck, sealing off the aneurysm from its parent vessel while preserving the normal blood flow [17].

Although nowadays minimally invasive methods are preferred [23], there are still some technically difficult cases where surgical intervention is necessary [6]. Consequently, the few aneurysms that have to be treated by clipping are usually complex. The resulting lack of practical experience in combination with the complexity of the cases is a huge problem for novice neurosurgeons, increasing the demand for more training possibilities.

The aim of this work was to develop an accessible and cheap desktop simulation tool with focus on visualising distance and force information to guide the surgeon. The distance visualisations display distances between the clip and vessel surface, aiming to enhance spatial relations and to improve the navigation of the clip. The user as well as the vessels remains at their initial positions, whereas the clip is moved. Since the clip is applied to the vessels, it is important to know where it is located in relation to the vessel surface. Therefore, the visualisations display the distances between the clip and the vessel surface. This visual support is necessary, as the application renders a three-dimensional (3D) scene on a two-dimensional (2D) monoscopic display.

The force visualisations display the force acting on the vessel surface caused by the applied clip. Depending on the chosen clipping method, more or less stress acts on the vessel, possibly leading to injuries and rupture during surgery [16, 41]. Thus, with our simulation tool, surgeons and trainees can try different methods and use the force feedback to evaluate them. In clinical practice, this is not possible. Nevertheless, it is important to keep in mind that there are many other important factors influencing the clip placement like preventing parent vessel stenosis or occlusion of surrounding vessels and perforating arteries [5]. Furthermore, force visualisations can replace the missing haptic feedback while closing the clip.

In summary, we presented a desktop application for aneurysm clipping with visualisations, facilitating the navigation and providing more feedback. Consequently, the whole training process as well as preoperative planning is supported.

## Related work

There are already a few approaches attempting to provide appropriate training by clipping simulations. That is why the first part of the related work focuses on aneurysm clipping simulations, whereas the following parts focus on distance and force visualisations in different medical contexts.

To provide surgeons and trainees with additional training, several simulation tools have been developed in the last years [1, 13, 24, 31, 37, 38]. Mashiko et al. [24] created a physical hollow elastic model, whereas Alaray et al. [1], Gmeiner et al. [13], Shono et al. [31] and Vite et al. [38] developed virtual clipping simulations. Vite et al. [37] also proposed a hybrid simulation.

Many approaches aim to develop tools that are as realistic as possible and thus intended to create realistic interactions by providing haptic feedback [1, 13, 37, 38]. However, they have in common that the users were not satisfied with the haptic feedback. One important task of haptic feedback is knowing when the executor is touching something; otherwise, it would be difficult to know when the clip is touching the aneurysm or a vessel.

Instead of using haptic feedback, we focus on providing other or additional guidance. As we do not intend to develop a simulation that is as realistic as possible, but to provide a training possibility, additional guidance in the form of visualisations is an appropriate alternative.

### Distance visualisations

As mentioned above, the haptic feedback provided in previous approaches is rated as not appropriate. To better support the users with guidance revealing the clip location and spatial relations, one type of information we visualise is distance information. Distance feedback is not available during microsurgical clipping, but it can help preparing for such a procedure, help trainees to get a better feeling and understanding and support the navigation.

There are several approaches in the medical simulation field, for which displaying distance, usually between a tool and target anatomy, is relevant. Kersten-Oertel et al. [20] summarised different ways of visualising distances. One possibility is to display the distance numerically [12, 34], or showing it numerically in a bar graph [19]. Another common way is to use a colour-coding scheme, lighting up the specific anatomy [18]. Colour-coded distance fields displayed on an anatomical structure are often used [7, 35]. Preim et al. [28] used coloured vessels to reveal different security margins that support the planning of liver surgery. Dick et al. [7] compared a glyph-based visualisation with a slice-based visualisation with colour-coded distances for interactive preoperative implant planning. Alternatively, Hansen et al. [14] propose a distance-encoding silhouette instead of distance-encoding surface. Another possibility is to use additional geometric structures like Trevisan et al.’s [39] dynamic sphere that changes its location according to the instrument’s position and its colour according to the distance to the risk structure. Due to the more complex situation in oncological pelvic surgical planning, Smit et al. [33] decided to visualise distances by colour mapping and isolines.

In case of endoscopy and image-guided surgery, it is useful to display the distance between the instrument and the target or risk structure. Winne et al. [42] used a guiding line during endoscopy, indicating the distance of the target and pointer instrument by changing its colour every millimetre. Heinrich et al. [15] also introduced a colour-coded pointer ray from the tip of the instrument aligned with the tool axis. To detect surrounding structures, a side-looking radar, rotating around the instrument’s tip, was developed. Another method they proposed is a virtual lighthouse. The users can place the lighthouses at predefined anatomical landmarks, from where rays towards the instrument tip are emitted.

Distances can be represented not only in 3D models, but also in medical volume data using transfer functions [36]. Although there are several approaches to visualise distances in medical applications, they have to be carefully chosen depending on specific tasks and related requirements.

### Force visualisations

In our simulation tool, the force that is displayed is obtained by a *mass–spring model*. Therefore, for each surface point the magnitude and direction of displacement that is caused by the force applied by the clip can be visualised. Consequently, we considered related work with respect to visualisations of stresses and tensor fields in connection with aneurysms, but also in other medical contexts.

Visualisations related to aneurysms are mostly used to either show the blood flow [11] or other hemodynamic information like wall-shear stress [30]. Meuschke et al. [25] proposed a combined visualisation of vessel deformation and hemodynamics. One concept is to unfold the aneurysm dome and visualise the deformation as colour map, and additional bars can visualise any scalar parameter, for example, wall thickness. Another combination was to depict the wall deformation as a colour map on the aneurysm surface and additional isolines showing the wall-shear stress. Meuschke et al. [26] compared four glyph-based tensor stress visualisation techniques for cerebral aneurysms. Here, glyphs were chosen that should show the main directions of the stress tensors as well as the corresponding stress values along the main directions. The four compared glyphs were superquadrics, kite-shaped glyphs, streamlines and scatterplots. The mentioned approaches all visualise stresses and forces during the cardiac cycle, but there is no approach combining such visualisations in an aneurysm clipping simulation.

Another approach of stress tensor visualisation in the medical field was proposed by Dick et al. [8]. They introduced an interactive visualisation of stress tensor fields supporting the planning of hip joint replacement. Their approach is one of the first approaches allowing interactive visual exploration of time-varying 3D stress tensor fields.

## Simulation workflow

**Fig. 1:**
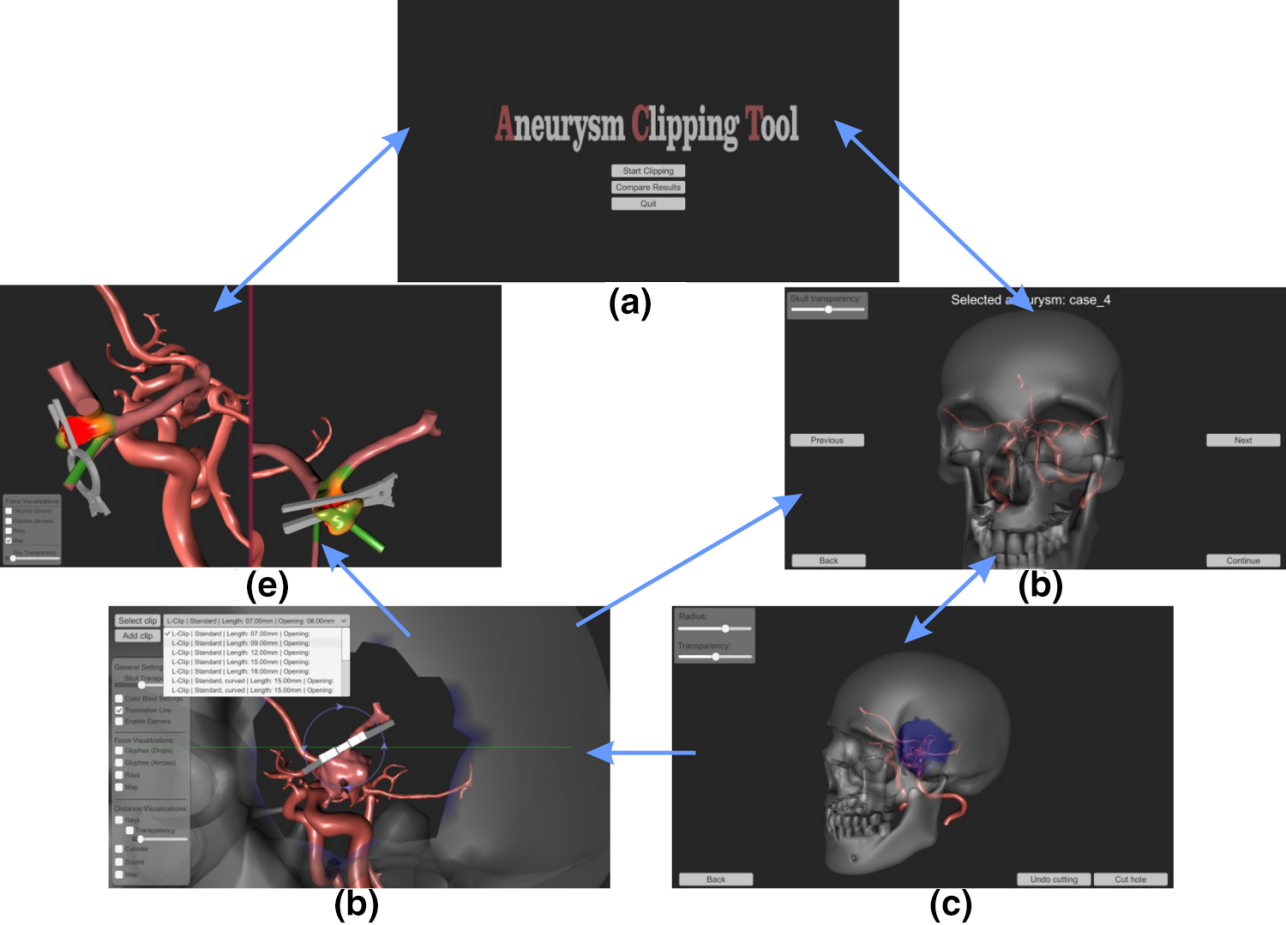
Overview of the simulation tool. Blue arrows illustrate the transitions between the scenes: **a** start scene, **b** Aneurysm selection, **c** craniotomy, **d** clipping process and **e** comparing two results

The whole simulation was implemented in Unity and comprises the scenes displayed in Fig. 1. After the start scene, the user has to select a predefined aneurysm. We decided not to use patient-specific models. Instead of this, aneurysms were modelled specifically to create a clinically relevant and representative set of cases based on a healthy Circle of Willis (CoW). We extracted a CoW from a healthy patient’s MRI data with a voxel resolution of .26 $\times$ .26 $\times$ .5 mm with our customised workflow [29]. The aneurysms were constructed with the expertise of a senior physician of neurosurgery and according to Kumar et al.’s [21] classification of M1, M2 bifurcation *middle cerebral artery (MCA) aneurysms*. This classification is based on morphological features which influence the techniques of clipping. These aneurysms are the most common MCA aneurysms [10] and are usually treated by clipping [21]. So it is ensured that the simulation provides a selection of five appropriate and relevant aneurysms. To enable a wider variety of aneurysms and to have the possibility of training with patient-specific data, the user can also import segmented aneurysm models.

The selected aneurysm is then shown in the next scene, the craniotomy. Here, the user has to select the location and radius of the hole. This hole is used as starting position for the clipping procedure. As in a real surgery, the user has a variety of clips and can apply one clip after the other. The eighteen clip models were constructed based on a product catalogue of the clip company (Peter Lazic GmbH Microsurgical Innovations, Tuttlingen, Germany; https://www.lazic.de/).

## Vessel deformation

By applying a clip at the aneurysm, the vessels are deformed according to the applied force. The deformation is simulated based on a mass–spring model and the *Verlet integration*. A mass–spring model was chosen as this method is not that computationally expensive and still provides a plausible deformation. Mass points are the vertices of the vessels triangle mesh which are displaced based on the calculations.

First, spring forces are determined according to *Hooke’s law*:

1 $$\begin{aligned} f_\mathrm{ij}^{t} = k_\mathrm{s} \times \frac{x_\mathrm{j} - x_\mathrm{i}}{|x_\mathrm{j} - x_\mathrm{i}|} \times \left( |x_\mathrm{j} - x_\mathrm{i}| - l_\mathrm{ij}^{0}\right) \end{aligned}$$

where $f_\mathrm{ij}^{t}$ is the spring force of the spring at time step *t* between mass i and j, with their positions $x_\mathrm{i}$ and $x_\mathrm{j}$, $k_\mathrm{s}$ is the spring constant of this spring, and $l_\mathrm{ij}^{0}$ is the rest length. The resulting force is applied equally and oppositely to both points. After calculating the spring forces, the next position of each mass point is determined by numerically solving the differential equation with the given force *f* and mass *m*: $f(t)/m(t)=\frac{\mathrm{d}x(t)}{\mathrm{d}t}$. This is done according to Eq. 2

2 $$\begin{aligned} x_{t+ \Delta t} = x_\mathrm{t} + (x_\mathrm{t} - x_{t-\Delta t}) \times (1 - k_\mathrm{d}) + \frac{f(t)}{m} \times \Delta t \times \Delta t \nonumber \\ \end{aligned}$$

where $x_{t-\Delta t}$ is the previous position and $k_\mathrm{d}$ is the damping constant. However, a mass point is only set to this new position if no collision takes place, resulting in the following two cases:

3 $$\begin{aligned} x_{t+ \Delta t, \mathrm{adapted}} = {\left\{ \begin{array}{ll} x_{t} + (x_{t+ \Delta t} - x_{t}) \times (\mathrm{d}_\mathrm{collision} \times 0.9) ,&{} \text {if colliding} \\ x_{t+ \Delta t},&{} \text {otherwise} \end{array}\right. } \end{aligned}$$

In the case of a collision, the next position is set to a point directly in front of the collision point by moving it by 0.9 times the collision distance $d_\mathrm{collision}$. If there is no collision, the next position equals the resulting position of the Verlet integration $x_{t+ \Delta t}$. The last step in the deformation iteration aims to compensate errors due to too large time steps. To avoid these errors, a stretching compensation as proposed by Duan et al. [9] was included.

In a mass–spring model, the parameters, such as the spring constant, are not obvious [22]. They were set such that the deformation is plausible and the calculation error is minimised. Regarding an expert, neurosurgeons do not require a perfectly realistic deformation, as they know how the vessels usually deform. Instead it is sufficient to have a plausible deformation. The plausibility of our deformation was confirmed by the expert.

## Visualisations

All distance and force visualisation concepts employ an underlying colour scale. Colour scales can be divided into three categories: qualitative, sequential and diverging [3]. Diverging maps are used when a significant value near the median is represented [27]. Two different colours highlight the zero-crossing in the data [32]. Using sequential maps, mostly a scalar value is mapped to saturation, showing the order of the data [27, 32]. Qualitative colour maps, on the other hand, are used to represent nominal data, which can be categorised, but not ordered [27, 32]. The underlying distance and force data are sequential. However, the visualisations do not aim to display the exact values, but a classification into low, mid-range and high values. This order and division into three parts can be achieved with a diverging colour scale when using appropriate colours [27]. Consequently, a diverging colour scale is used even if there is no zero-crossing in the data. Based on these three regions, a green-to-red colour scale was chosen, as these colours are intuitively associated with critical and non-critical situations. Consequently, large distances and low magnitudes are represented in green and low distances and high magnitudes in red, so in both cases the critical values are displayed in red.

One disadvantage of this map is that it is not usable for people with dyschromatopsia. Therefore, for these people a second colour scale ranging from red to blue is provided [27].

### Distance visualisations

**Fig. 2:**
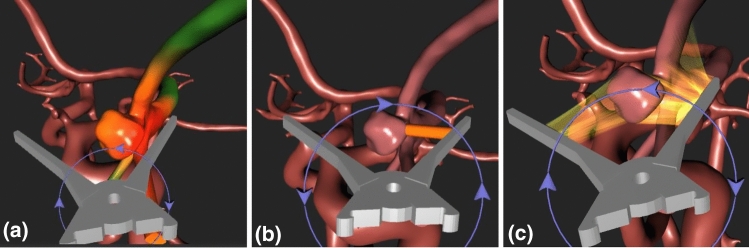
Distance visualisations showing the distance between the clip and the vessel surface. **a** Colour map, **b** cylinder and **c** semi-transparent rays

To visualise the distance between the clip and the vessels, three concepts were integrated in the simulation. First, a colour map is drawn on the vessel surface, resulting in the visualisation shown in Fig. 2a. To assess distances not just by their values, the next visualisations show the distances by highlighting the corresponding line. This results in a second concept displaying the smallest distance as a cylinder, see Fig. 2b. The last concept aims at not just highlighting the smallest distance, but all distances smaller than a specific threshold as semi-transparent rays, which is shown in Fig. 2c.

### Force visualisations

The force information is based on the mass–spring deformation and is approximated by the vector between the initial position of each vessel vertex and the current, possibly displaced position. Consequently, the direction and magnitude are available to visualise.

**Fig. 3:**
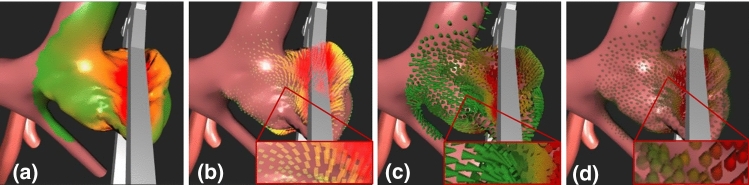
Force visualisations showing the magnitude and if possible the direction of the displacements of the vessel surface points that is caused by the applied clip. **a** Colour map, **b** semi-transparent rays, **c** arrow glyphs and **d** drop glyphs

The first concept is a colour map, see Fig. 3a, displaying the magnitude by colour. The second concept, shown in Fig. 3b, draws for each vertex of the vessel’s mesh a semi-transparent ray from its initial position to its current position. Hence, the magnitude is displayed by length and the direction by orientation. Furthermore, two glyph-based concepts were integrated and are displayed in Fig. 3c, d. The first glyph is an arrow showing the magnitude by colour and the direction by its orientation. The glyph for the second visualisation has the shape of a drop and colour-codes the strength and orientation like the glyph before. Additionally, this glyph is scaled along its main axis according to the strength, resulting in longer drops representing strong displacements. To prevent strong displacements from occluding smaller ones, scaling the glyphs only takes place within a certain range. Furthermore, the glyphs are drawn semi-transparently corresponding to the strength. Both additional features ensure that strong displacements are more highlighted than small ones. The shape of a drop arose from the common flow visualisation technique oriented line integral convolution (OLIC) [40]. Also, drops are a naturally occurring shape, so they are easy to understand in terms of their orientation.

## Evaluation

To evaluate the simulation tool and its visualisation concepts, a quantitative and a qualitative evaluation was performed. The former was done by an online survey with five male and six female participants. Four of them are practicing neurosurgeons, and the others are medical students shortly before graduation, meaning they have previous knowledge in the area of neurosurgery. All of them were familiar with cerebral aneurysms and clipping beyond the textbook because of their final assignments or field work in this area. Therefore, their answers have a certain relevance. Two of the neurosurgeons have one to five years of experience, and the other two have six to ten years of experience.

The focus of the online survey was on assessing the force visualisations, as they can be evaluated based on pictures. To evaluate the distance visualisations properly, experts have to interact with the tool and navigate the clip on their own. This was done during the qualitative evaluation. Here, one neurosurgeon with four years of experience had to interact with the tool and the task to try the different distance visualisations while navigating the clip towards the aneurysm. Hereby, he was asked to think aloud and was encouraged to comment on the force visualisations.

In the survey, the participants had to rate different statements with a 5-point Likert scale for each individual concept. With the statements, we wanted to understand whether:

- They would use the visualisation,
- All information is recognisable,
- No important information is occluded,
- The visualisation provides added value,
- The visualisation is intuitive.

**Fig. 4:**
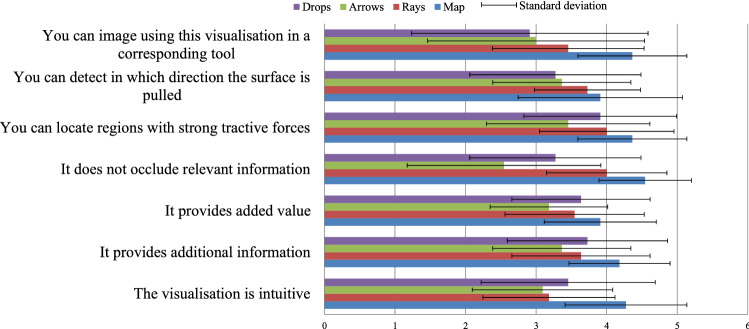
Average scoring for the force concepts regarding the different statements and the corresponding standard deviation

**Fig. 5:**
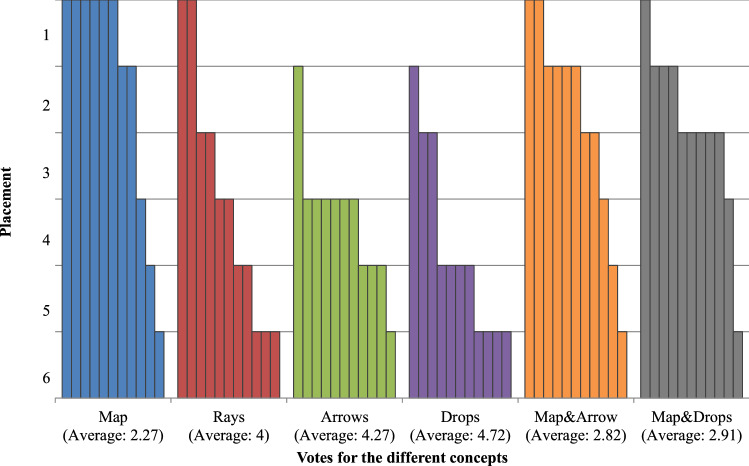
Ranking of the force concepts. Each bar represents a chosen rank by a participant for the specific concept

The exact statements and their assessment can be seen in Fig. 4. After assessing each concept, the experts had to rank them. Additionally to the presented concepts, the ranking also comprises two combinations of the colour map and the two glyphs. The rankings are shown in Fig. 5. The statements as well as the ranking of the colour map achieve the highest scores. Thus, the colour map is the best concept, providing the user with an appropriate additional force information. This visualisation was the distinct favour of the expert during the qualitative evaluation, too. As he stated, this visualisation is a good compensation for the lack of haptic feedback.

The results of the distance visualisations are based on the qualitative evaluation. The first important insight is that in this case, i.e. a desktop application on a monoscopic display, distance visualisations are required to navigate the clip properly. This is because there are no binocular depth cues available and some monocular depth cues like texture gradient are missing, too. The lack of several depth cues in such a context complicates the depth perception. Without a visualisation, it was not possible for the expert to estimate the depth, resulting in closing the clip unintentionally multiple times before reaching the vessels. Only the semi-transparent rays gave enough information, thus supporting the navigation properly.

During the qualitative evaluation, the tool was evaluated according to three criteria: usability, realism and performance. Regarding usability, the expert stated that the whole workflow is clear and intuitive. The interaction via keyboard is appropriate, but the clip movement can be improved by having a free axis along which the clip is moved instead of three fixed axes.

As the clip models are based on measurements given in a product catalogue and the aneurysm models are constructed based on the knowledge and instructions of an expert, they can be rated as realistic and relevant. According to the expert, not just the models but also the deformation is plausible and appropriate enough for such a clipping simulation.

The performance was satisfying. For the evaluation, a laptop was used (Alienware, NVIDIA GeForce GTx 1080 with Max-Q Design, intel core i7-7820HK CUP 2.9GHz, 2901 MHz, 4 cores, 8 logical processors).

## Conclusion

We presented a prototype of an easily accessible clipping simulation for unruptured cerebral aneurysms that does not require any expensive hardware. As the simulation comprises realistic models of different clips and aneurysms, it provides an appropriate training possibility for neurosurgeons and trainees. With our prototype, it is possible to enhance such a clipping simulation by integrating distance and force visualisations. More precisely, semi-transparent rays are able to support the navigation of the clip, whereas a colour map is appropriate for assessing different clipping methods.

To finally provide this simulation to neurosurgeons and trainees, the visualisations and the whole tool can be refined based on further evaluations with more experts. When evaluating with more experts, a comparable scale like the NASA TLX should be used. Regarding the integrated clip models, an improvement could be to provide the possibility for including other and more clips. Additionally, the simulation can be enhanced by adding the exposure of the aneurysm to the workflow. Furthermore, refinements regarding the two preferred visualisations, the interaction and deformation can be made. Generally, the distance visualisation is not limited to cerebral aneurysms and can be easily adapted to similar simulations, including navigation.

Consequently, the two visualisations enhance a clipping simulation and thus improve a possible training environment for neurosurgeons and trainees.
